# Six-Month Outcome of Transient Ischemic Attack and Its Mimics

**DOI:** 10.3389/fneur.2019.00294

**Published:** 2019-03-27

**Authors:** Alireza Sadighi, Vida Abedi, Alia Stanciu, Nada El Andary, Mihai Banciu, Neil Holland, Ramin Zand

**Affiliations:** ^1^Department of Neurology, Geisinger Medical Center, Danville, PA, United States; ^2^Department of Bioinformatics, Geisinger Medical Center, Danville, PA, United States; ^3^Freeman College of Management, Bucknell University, Lewisburg, PA, United States

**Keywords:** DWI-negative transient ischemic attack, transient ischemic attack mimics, DWI-positive transient ischemic attack, composite outcome, follow-up study

## Abstract

**Background and Objective:** Although the risk of recurrent cerebral ischemia is higher after a transient ischemic attack (TIA), there is limited data on the outcome of TIA mimics. The goal of this study is to compare the 6-month outcome of patients with negative and positive diffusion-weighted imaging (DWI) TIAs (DWI-neg TIA vs. DWI-pos TIA) and also TIA mimics.

**Methods:** We prospectively studied consecutive patients with an initial diagnosis of TIA in our tertiary stroke centers in a 2-year period. Every included patient had an initial magnetic resonance (MR) with DWI and one-, three-, and six-month follow-up visits. The primary outcome was defined as the composition of intracerebral hemorrhage, ischemic stroke, TIA, coronary artery disease, and death.

**Results:** Out of 269 patients with the initial diagnosis of TIA, 259 patients (mean age 70.5 ± 15.0 [30–100] years old, 56.8% men) were included in the final analysis. Twenty-one (8.1%, 95% confidence interval [CI] 5.1-12.1%) patients had a composite outcome event within the six-month follow-up. Five (23.8%) and 13 (61.9%) composite outcome events occurred in the first 30 and 90 days, respectively. Among patients with DWI-neg TIA, the one- and six-month ischemic stroke rate was 1.5 and 4.6%, respectively. The incidence proportion of composite outcome event was significantly higher among patients who had the diagnosis of DWI-neg TIA compared with those who had the diagnosis of TIA mimics (12.2 vs. 2.1%—relative risk 5.9; 95% CI, 1.4–25.2). In our univariable analysis among patients with DWI-neg TIA and DWI-pos TIA, age (*P* = 0.017) was the only factor that was significantly associated with the occurrence of the composite outcome.

**Conclusion:** Our study indicated that the overall six-month rate of the composite outcome among patients DWI-neg TIA, DWI-pos TIA, and TIA mimics were 12.2, 9.7, and 2.1%, respectively. Age was the only factor that was significantly associated with the occurrence of the composite outcome.

## Introduction

After a transient ischemic attack (TIA), the risk of ischemic stroke is high and ranging from 9 to 20% within the first 3 months (1–3). Patients suffering from TIA also have higher risks of myocardial infarction (4), disability, and death (5, 6). A long-term outcome study of patients with TIA has shown that the 10-year risk of stroke can be up to 19% while combined outcome risks including stroke, myocardial infarction, and death can be as high as 43% (7).

Although there is not a widely accepted definition for TIA mimics and there is only limited number of studies on patients with TIA mimics (3, 8), reports are indicating that more than 50% of patients who are referred to TIA clinics are TIA mimics (9, 10). Patients who are classified as TIA mimics have the heterogeneous etiologies ranging from cardiac ischemic events, dementia, strokes, to benign conditions (11, 12).

There is limited evidence on the outcome of TIA mimics. At the same time, some of the previous TIA outcome studies used the TIA clinical definition that alone cannot differentiate between a DWI-neg (DWI: diffusion-weighted imaging, neg: negative) TIA and DWI-pos (pos: positive) TIA. In this prospective study, we aimed to assess and compare the 6-month outcome of patients with DWI-neg TIA and TIA mimics, as well as patients with DWI-pos TIA.

## Materials and Methods

We prospectively studied consecutive patients with an admission diagnosis of TIA in one of Geisinger three tertiary stroke centers or referral TIA diagnosis in our single TIA clinic in northeast Pennsylvania in a 2-year period (2016–2018). Geisinger is a comprehensive healthcare system with several hospitals and clinics, and over one million active patients of around 90% white ethnicity in central and northeast Pennsylvania, as well as southeast New Jersey.

### Patient Inclusion Criteria

Included patients in our study had the admission diagnosis of TIA in one of our three tertiary stroke centers or had been referred to our dedicated TIA clinic with the referral diagnosis of TIA. Every included patient had an interpretable initial brain magnetic resonance imaging (MRI). Each patient had presented with a transient focal neurological deficit that lasted <24 h. All hospitalized patients were evaluated by an attending neurologist within 24 h. Every patient had either follow-up visits at our TIA/stroke clinic or phone encounter in the following intervals: 1 month, three, and 6 months ± 7 days following the index TIA event. We excluded patients who missed to complete the 6-month follow-up course.

### Diagnostic Process

The final diagnoses of DWI-neg TIA, TIA mimics, or DWI-pos TIA were made independent of the hospital discharge and clinic diagnoses at the end of a 6-month follow-up period. Although there is no clear definition for TIA mimics, we concluded patients with “TIA mimics” when the diagnosis of TIA was ruled out, and a different diagnosis (e.g., seizure, migraine headache) was made. Patients with “DWI-neg TIA” diagnosis included patients who had no alternative diagnosis or patients with probable TIA diagnosis with negative findings in DWI. Patients with the diagnosis of “DWI-pos TIA” had their symptoms resolved within 24 h; however, they had a positive DWI for acute ischemic stroke. We independently reviewed each case, and final diagnoses were made based on all the clinical information after the completion of all follow-ups and consensus between our stroke research fellow (AS), and one of our vascular neurologists (NE). In the absence of a diagnostic consensus, our second vascular neurologist (RZ) reviewed the patient medical record and acted as a tiebreaker.

### Outcome Measures

The primary outcome was defined as the composition of intracerebral hemorrhage (ICH), ischemic stroke, TIA, coronary artery disease (CAD), and all-cause death. Patients had an ischemic stroke outcome when they presented with a focal neurological deficit and a confirmatory cerebral MRI or CT Scan. All cases of recurrent stroke and DWI-neg TIA were concluded. Patients with ICH had confirmatory cerebral MRI or CT Scan, as well. Patients with CAD had signs of typical chest pain confirmed by ST-T wave changes in ECG or high blood troponin levels. We also reviewed cardiology notes for further confirmation. We calculated total composite outcome events for the studied cohort in the following intervals: 30, 90, and 180 days ± 7 days following the index event. We recorded all the patients' medical record information including demographics, presenting symptoms, past medical history, medication list, clinical work-up, and imaging study results. We calculated ABCD^2^ risk score for every patient (13). We also reviewed DWI, FLAIR, T2^*^-weighted gradient recalled echo (GRE) sequences, and CT scan. We applied Fazekas scale for white matter lesions scoring (14, 15). This study was part of the ongoing Geisinger stroke registry and approved by institutional review board of Geisinger.

### Statistical Analysis

We summarized all continuous variables as mean ± standard deviation (normal distribution) and as median with Inter Quantile Range (IQR, for skewed distribution). We summarized all categorical variables as percentages with their corresponding 95% Confidence Intervals. We applied Kruskal–Wallis test for continuous variables. We performed statistical comparisons between three groups using the χ2 test or, in the case of small expected frequencies, Fisher's exact test. We performed multivariable analysis of logistic binary regression model. We performed Kaplan-Meier survival analysis to evaluate “time-to-composite outcome event” information and also the Log-Rank statistical test was applied for evaluation of differences among studied groups (*P* < 0.05). We used SPSS 24.0 (Chicago, Ill., USA) for all our statistical analysis. Incidence rates were calculated using MedCalc software (16).

## Results

**Figure 1:**
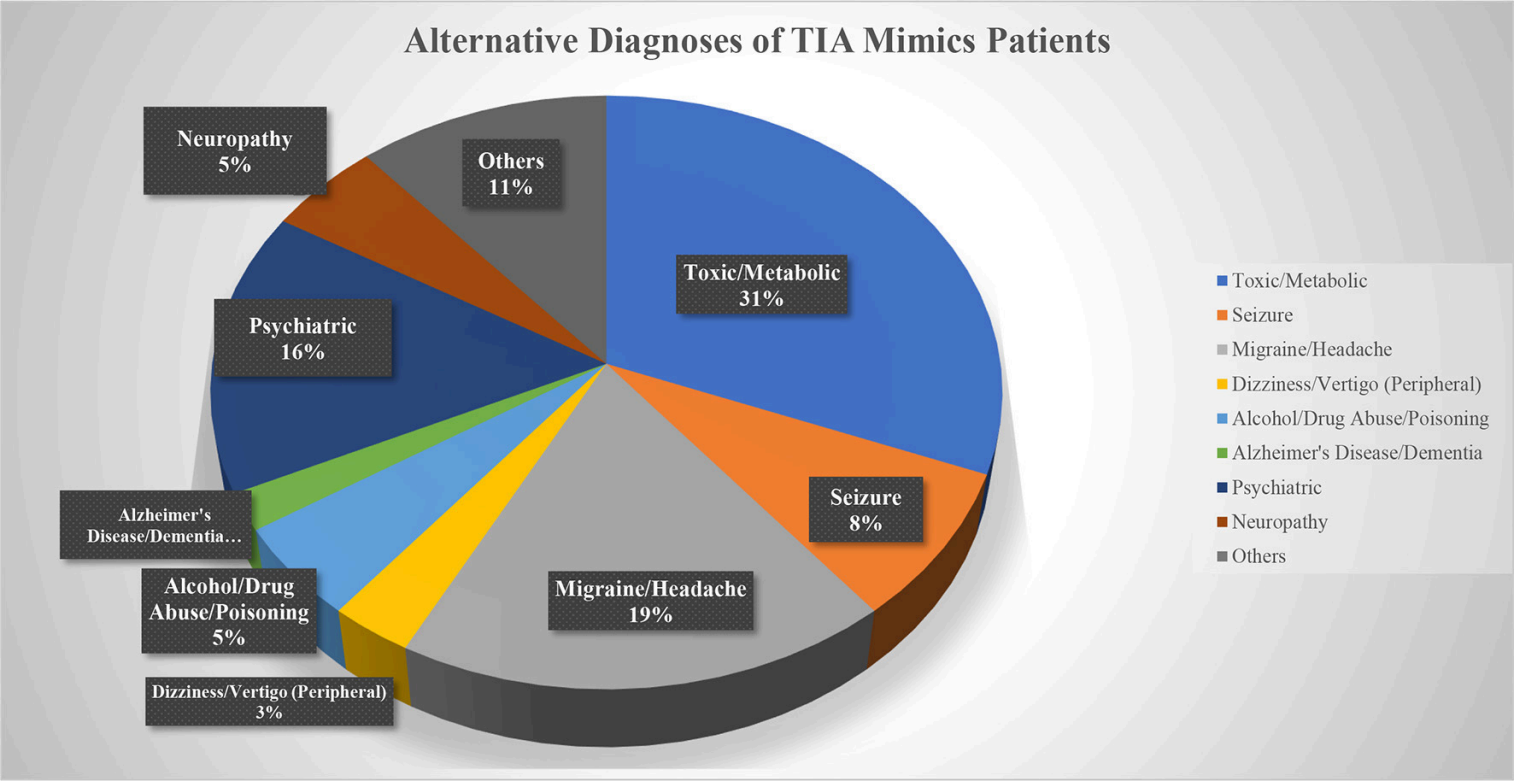
Alternative diagnoses of TIA Mimics patients.

Out of 269 patients with the initial diagnosis of TIA, 259 patients (mean age 70.5 ± 15.0 [30–100] years old, 56.8% men) were included in the final analysis (Table 1). Ten patients (2 patients with a diagnosis of TIA mimics and 8 patients with a diagnosis of DWI-neg TIA) who missed to complete 180 days follow-up course were excluded. Out of 259 patients, 97 (37.4%) patients had the diagnosis of TIA mimics, 131 (50.6%) patients had a DWI-neg TIA diagnosis, and 31 (12.0%) patients had the diagnosis of DWI-pos TIA. Carotid endarterectomy was performed only for three (1.2%) patients in the group of DWI-neg TIA. The most common alternative diagnosis in the TIA mimics group was toxic/metabolic diseases followed by migraine (Figure 1).

**Table 1:** Univariate analysis comparing TIA Mimics, DWI-neg TIA, and DWI-pos TIA groups.

	**^**$**^TIA mimics group *N* = 97 (37.5%)**	**DWI-neg TIA group *N* = 131 (50.6%)**	**DWI-pos TIA group *N* = 31 (12.0%)**	**Total (*N* = 259)**	***P*-Value**
**Demographic information**					
Gender (% of males)	63 (64.9%)	73 (55.7%)	11 (35.5%)	147 (56.8%)	0.015^*^
Age, mean ± SD	64.6 ± 17.0	73.7 ± 13.1	74.8 ± 11.0	70.5 ± 15.0	0.000^*^
Race					
White	94 (96.9%)	128 (97.7%)	30 (96.8%)	252 (97.3%)	0.934
African-American	2 (2.1%)	3 (2.3%)	1 (3.2%)	6 (2.3%)	
Patient declined to provide	1 (1.0%)	0 (0.0%)	0 (0.0%)	1 (0.4%)	
**Past medical history**					
Diabetes mellitus	26 (26.8%)	45 (34.4%)	9 (29.0%)	80	0.462
Hypertension	60 (61.9%)	113 (86.3%)	26 (83.9%)	199	0.000^*^
Atrial fibrillation, PAF^†^, flutter	9 (9.3%)	26 (19.8%)	8 (25.8%)	43	0.036^*^
Hyperlipidemia	66 (68.0%)	117 (89.3%)	27 (87.1%)	210	0.000^*^
Previous history of stroke	19 (19.6%)	35 (26.7%)	9 (29.0%)	63	0.375
Previous history of TIA	27 (27.8%)	35 (26.7%)	6 (19.4%)	68	0.637
Peripheral vascular disease	6 (6.2%)	20 (15.3%)	5 (16.1%)	31	0.085
Coronary artery disease (CAD)	29 (29.9%)	61 (46.6%)	14 (45.2%)	104	0.033^*^
Patent foramen ovale	7 (21.2%)	14 (22.2%)	2 (14.3%)	23	0.803
Intracranial arterial disease	16 (25.0%)	44 (48.9%)	13 (50.0%)	73	0.007^*^
COPD^‡^	14 (14.4%)	28 (21.4%)	5 (16.1%)	47	0.386
Carotid disease					
<50% Stenosis	43 (44.3%)	66 (50.4%)	14 (45.2%)	123	0.011^*^
50–70% Stenosis	6 (6.2%)	17 (13.0%)	5 (16.1%)	28	
>70% Stenosis	0 (0.0%)	7 (5.3%)	3 (9.7%)	10	
Tobacco use	22 (22.7%)	31 (23.7%)	3 (9.7%)	56	0.224
Alcohol use	25 (25.8%)	43 (32.8%)	8 (25.8%)	76	0.461
Seizure disorder	10 (10.3%)	1 (0.8%)	0 (0.0%)	11	0.001^*^
Migraine	20 (20.6%)	10 (7.6%)	2 (6.5%)	32	0.007^*^
Autoimmune disease	0 (0.0%)	5 (3.8%)	0 (0.0%)	5	0.083
Coagulation disorders	5 (5.2%)	7 (5.3%)	1 (3.2%)	13	0.886
History of cancer	17 (17.5%)	31 (23.7%)	9 (29.0%)	57	0.327
Kidney disease	19 (19.6%)	32 (24.4%)	8 (25.8%)	59	0.630
Currently on dialysis	0 (0.0%)	1 (0.8%)	0 (0.0%)	1	0.612
Psychiatric illness	48 (49.5%)	44 (33.6%)	7 (22.6%)	99	0.008^*^
Antiplatelet use (pre-hospitalization)	49 (50.5%)	86 (65.6%)	21 (67.7%)	156	0.046^*^
Oral anticoagulant (pre- hospitalization)	7 (7.2%)	16 (12.2%)	3 (9.7%)	26	0.462
**Emergency department presentation symptoms**					
Altered mental status	16 (16.5%)	29 (22.1%)	5 (16.1%)	50	0.505
Headache	25 (25.8%)	23 (17.6%)	7 (22.6%)	55	0.319
Loss of consciousness	3 (3.1%)	1 (0.8%)	1 (3.2%)	5	0.385
Generalized weakness	11 (11.3%)	13 (9.9%)	6 (19.4%)	30	0.335
Unilateral arm weakness	26 (26.8%)	32 (24.4%)	12 (38.7%)	70	0.273
Unilateral leg weakness	23 (23.7%)	26 (19.8%)	9 (29.0%)	58	0.504
Expressive aphasia	15 (15.5%)	28 (21.4%)	8 (25.8%)	51	0.356
Dysarthria	23 (23.7%)	51 (38.9%)	11 (35.5%)	85	0.051
Facial droop	14 (14.4%)	29 (22.1%)	7 (22.6%)	50	0.306
Unilateral arm numbness	43 (44.3%)	40 (30.5%)	9 (29.0%)	92	0.071
Unilateral leg numbness	23 (23.7%)	23 (17.6%)	7 (22.6%)	53	0.498
Facial numbness	26 (26.8%)	42 (32.1%)	3 (9.7%)	71	0.042^*^
Sudden true vertigo	9 (9.3%)	2 (1.5%)	0 (0.0%)	11	0.007^*^
Diplopia	1 (1.0%)	4 (3.1%)	0 (0.0%)	5	0.387
Hemianopsia	19 (19.6%)	12 (9.2%)	3 (9.7%)	34	0.058
Mono-ocular blindness	0 (0.0%)	2 (1.5%)	1 (3.2%)	3	0.294
Ataxia	13 (13.4%)	17 (13.0%)	5 (16.1%)	35	0.898
Seizure like activity	3 (3.1%)	1 (0.8%)	1 (3.2%)	5	0.385
Pre-syncope	13 (13.4%)	21 (16.0%)	6 (19.4%)	40	0.702
Visual Aura	4 (4.1%)	3 (2.3%)	0 (0.0%)	7	0.429
Amnesia	6 (6.2%)	9 (6.9%)	3 (9.7%)	18	0.800
Length of hospitalization (days), mean ± SD	1.9 ± 1.8	2.0 ± 1.5	4.0 ± 2.3	2.2 ± 1.8	0.000^*^
Duration of symptoms (minutes), mean ± SD	140.8 ± 258.2	191.7 ± 339.2	420.7 ± 481.7	207.9 ± 350.1	0.001^*^
**Imaging study results**					
WMD^¥^ on MRI Fazekas score					
Low	24 (29.3%)	42 (43.3%)	10 (33.3%)	76	0.006^*^
Moderate	22 (26.8%)	28 (28.9%)	10 (33.3%)	60	
Severe	12 (14.6%)	16 (16.5%)	9 (30.0%)	37	
^**§**^**ABCD**^**2**^ **Score**					
≤ 3	63 (64.9%)	58 (44.3%)	5 (16.1%)	126	0.000^*^
3 <	34 (35.1%)	73 (55.7%)	26 (83.9%)	133	
**Discharged patients' medications**					
Antiplatelet/anticoagulation therapy	77 (79%)	131 (100%)	31 (100%)	239	0.000^*^
Anti-cholesterol therapy	66 (68.0%)	117 (89.3%)	27 (87.1%)	210	0.000^*^
Antihypertensive therapy	60 (61.9%)	113 (86.3%)	26 (83.9%)	199	0.000^*^

$ *TIA, Transient ischemic attack*.

§ *ABCD^2^, (Age, Blood pressure, Clinical, Duration, Diabetes)*.

† *PAF, Paroxysmal atrial fibrillation*.

‡ *COPD, Chronic obstructive pulmonary disease*.

¥ *WMD, White matter disease*.

* *P < 0.05, Statistically meaningful*.

Among studied cohort (*N* = 259), the univariate analysis revealed a significant difference (*P* < 0.05) among the three groups (TIA mimics, DWI-neg TIA, and DWI-pos TIA) in terms of age, history of hypertension, atrial fibrillation, hyperlipidemia, coronary artery disease, intracranial arterial disease, psychiatric illness, carotid disease, and antiplatelet use (Table 1). Patients with DWI-pos TIA had longer symptoms' duration and higher ABCD^2^ score (Table 1).

**Figure 2:**
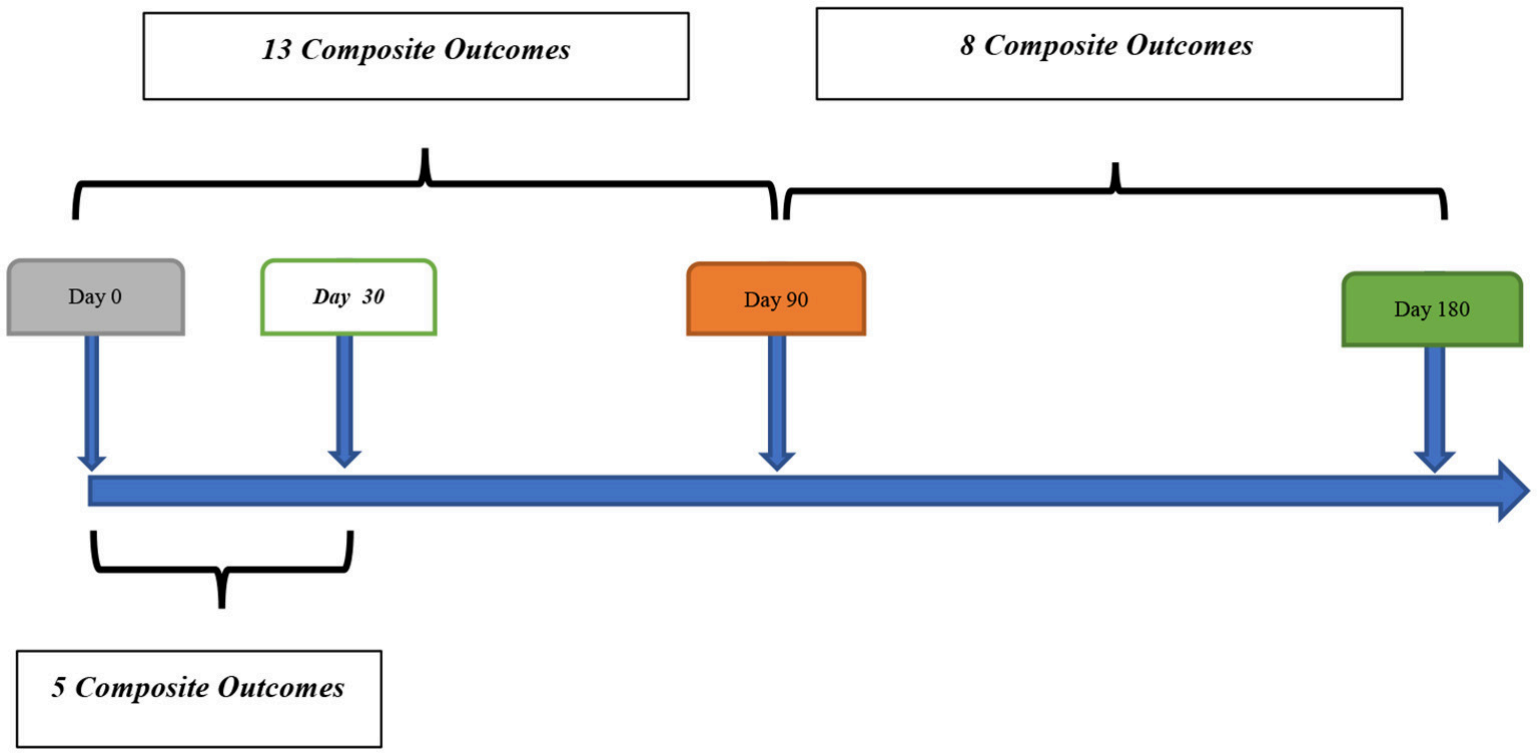
Timeline of composite outcomes.

Out of 259 patients, 21 (8.1%, 95% confidence interval [CI] 5.1–12.1%) patients had a composite outcome event within the 6-month follow-up. Although several patients had readmission for other reasons, we did not have any patient who had more than one composite outcome event during the 6 months follow-up. Five (23.8%) and 13 (61.9%) composite outcome events occurred in the first 30 and 90 days, respectively (Table 2). We also observed 8 (38.1%) composite outcome events in the second 90 days (Figure 2). Overall, patients with a diagnosis of DWI-neg TIA had a higher rate (12.2%) of composite outcome events followed by DWI-pos TIA (9.7%) and TIA mimics cohort (2.1%) (Table 3).

**Table 2:** Composite outcome events in early and late intervals.

	**30 days composite outcome events**	**First 90 days composite outcome events**	**Second 90 days composite outcome events**
Number of composite outcome events in TIA mimics patients (*N* = 2)	0	1	1
Number of composite outcome events in DWI-neg TIA patients (*N* = 16)	3	9	7
Number of composite outcome events in DWI-pos TIA patients (*N* = 3)	2	3	0
Total (*N* = 21)	5	13	8

**Table 3:** Outcome incidence proportions in 180 days follow-up course for studied cohort.

	^*^Incidence proportions in DWI-neg TIA patients	**Incidence proportions in TIA mimics patients**	**Relative risk**	**95% confidence interval**	**Incidence proportions in DWI-pos TIA patients**	**Incidence proportions in TIA mimics patients**	**Relative risk**	**95% confidence interval**	**Incidence proportions in DWI-neg TIA patients**	**Incidence proportions in DWI-pos TIA patients**	**Relative risk**	**95% confidence interval**
**OUTCOME**												
Composite outcome	12.2	2.1	5.9	1.4–25.2^**^	9.7	2.1	4.7	0.8–26.8	12.2	9.7	1.3	0.4–4.1
Ischemic stroke	4.6	0.0	9.7	0.6–169.3	3.2	0.0	9.2	0.4–220.0	4.6	3.2	1.4	0.2–11.4
Intracranial hemorrhage (ICH)	0.8	0.0	2.2	0.1–54.1	0.0	0.0	3.1	0.1–151.2	0.8	0.0	0.7	0.0–17.4
TIA	1.5	0.0	3.7	0.2–76.5	6.5	0.0	15.3	0.8–310.7	1.5	6.5	0.2	0.0–1.6
Coronary artery disease (CAD)	1.5	1.0	1.5	0.1–16.1	0.0	1.0	1.0	0.0–24.4	1.5	0.0	1.2	0.1–24.6
All cause death	3.8	1.0	3.7	0.4–31.2	0.0	1.0	1.0	0.0–24.4	3.8	0.0	2.7	0.2–47.0

* *All incidence proportions are in percentage*.

** *Statistically meaningful*.

### Outcome Events in Patients With the Diagnosis of TIA Mimics

**Figure 3:**
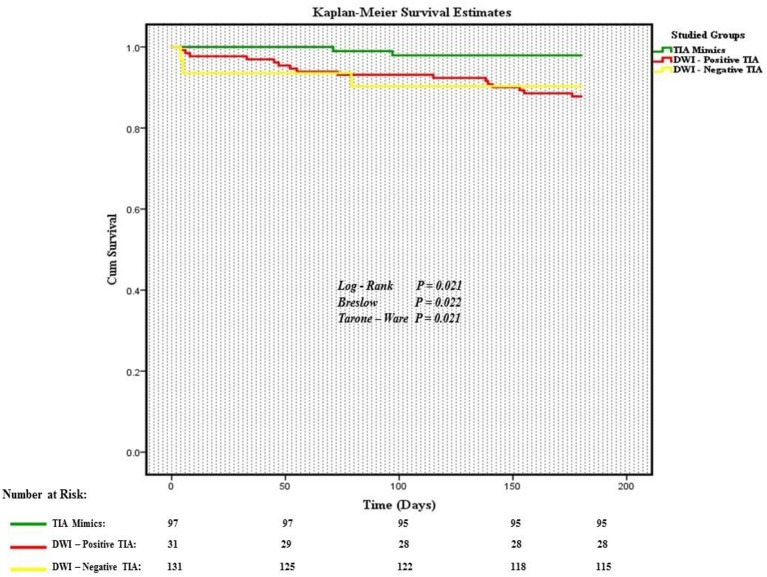
Kaplan-Meier estimates of composite outcomes.

The overall composite outcome rate was 2.1% (2 out of 97 patients) in this group. There was not any composite outcome event in the first 30 days after initial admission. There was only one composite outcome event (CAD) in the first 90 days followed by one composite outcome event of death due to cancer in the second 90 days (97th day) (Figure 3).

### Outcome Events in Patients With the Diagnosis of DWI-neg TIA

The overall composite outcome rate was 12.2% (16 out of 131 patients) in this group. More than half of the composite outcome events (9 events; 56.3%) occurred in the first 90 days. The single ICH outcome event occurred in this cohort. Our earliest outcome event (ischemic stroke) occurred on day 4. The one-month and six-month ischemic stroke rate was 1.5 and 4.6%, respectively. The one-month and six-month mortality rate were 0.0 and 3.8%, respectively (Table 3).

### Outcome Events in Patients With the Diagnosis of DWI-pos TIA

The composite outcome rate was 9.7% (3 out of 31 patients) among patients with DWI-pos TIA. There was no death in this group. We had one patient with TIA outcome event on day 4 after initial admission. We had only one (3.2%) patient with recurrent ischemic stroke outcome event (Table 3).

The incidence proportion of composite outcome event was higher among patients who had the diagnosis of DWI-neg TIA compared with those who had the diagnosis of TIA mimics (12.2 vs. 2.1%), the difference was significant (relative risk 5.9; 95% CI, 1.4–25.2). Although there was not a significant difference in the incidence proportion of composite outcome events between patients with the diagnosis of DWI-pos TIA and patients with TIA mimics (relative risk 4.7; 95% CI, 0.82–26.8), there was a trend for a higher rate of composite outcome events in patients with DWI-pos TIA (9.7 vs. 2.1%) (Table 3).

We performed a univariable analysis among patients with DWI-neg TIA and DWI-pos TIA (Table 4). Although we observed a higher rate of hypertension (*P* = 0.078), the severity of white matter disease (*P* = 0.076), and peripheral vascular disease (*P* = 0.082) among patients with the positive composite outcome, the difference was not significant. In our analysis, age (*P* = 0.017) was the only factor that was significantly associated with the occurrence of the composite outcome. We performed a multivariable binary logistic regression analysis for our composite outcomes based on univariable analysis results. All variables with *P* < 0.15 (Table 4) in the univariate model including: age, hypertension, peripheral vascular disease, intracranial arterial disease, seizure disorder, and white matter disease were included in the model. None of the variables including age was found to be significantly associated with the outcome.

**Table 4:** Univariate analysis: composite outcome and associated factors among patients with DWI-neg TIA and DWI-pos TIA.

	**Negative outcome event *N* = 143**	**Positive outcome event *N* = 19**	***P*-Value**
**Demographic Information**			
Gender (% of males)	72 (50.3%)	12 (63.2%)	0.336
Age, mean ± SD	73.1 ± 12.8	80.4 ± 8.5	0.017*
**Past medical history**			
Diabetes mellitus	45 (31.5%)	9 (47.4%)	0.198
Hypertension	120 (83.9%)	19 (100%)	0.078
Atrial fibrillation, PAF^†^, flutter	25 (20.3%)	5 (26.3%)	0.553
Hyperlipidemia	126 (88.1%)	18 (94.7%)	0.698
Previous history of stroke	36 (25.2%)	8 (41.2%)	0.167
Previous history of TIA	36 (25.2%)	5 (26.3%)	0.999
Peripheral vascular disease	19 (13.3%)	6 (31.6%)	0.082
Coronary artery disease (CAD)	64 (44.8%)	11 (57.9%)	0.332
Patent foramen ovale	14 (20.3%)	2 (25%)	0.668
Intracranial arterial disease	46 (46.0%)	11 (68.8%)	0.111
COPD^‡^	29 (20.3%)	4 (21.1%)	0.999
Carotid disease			
<50% Stenosis	69 (48.3%)	11 (57.9%)	0.191
50–70% Stenosis	18 (12.6%)	4 (21.1%)	
>70% Stenosis	8 (5.6%)	2 (10.5%)	
Tobacco use	30 (21.0%)	4 (21.1%)	0.999
Alcohol use	47 (32.9%)	4 (21.1%)	0.431
Seizure disorder	0 (0.0%)	1 (5.3%)	0.117
Migraine	10 (7.0%)	2 (10.5%)	0.635
Autoimmune disease	5 (3.5%)	0 (0.0%)	0.999
Coagulation disorders	8 (5.6%)	0 (0.0%)	0.598
History of cancer	33 (23.1%)	7 (36.8%)	0.255
Kidney disease	33 (23.1%)	7 (36.8%)	0.255
Currently on dialysis	1 (0.7%)	0 (0.0%)	0.999
Psychiatric illness	43 (30.1%)	8 (42.1%)	0.302
Antiplatelet use (pre-hospitalization)	93 (65.0%)	14 (73.7%)	0.608
Oral anticoagulant (pre-hospitalization)	17 (11.9%)	2 (10.5%)	0.999
**Imaging study results**			
WMD^¥^ on MRI Fazekas score			
Low	47 (43.1%)	5 (27.8%)	0.076
Moderate	32 (29.4%)	6 (33.3%)	
Severe	18 (16.5%)	7 (38.9%)	
^**§**^**ABCD**^**2**^ **score**			
≤ 3	58 (40.6%)	5 (26.3%)	0.318
3 <	85 (59.4%)	14 (73.7%)	

§ *ABCD^2^, (Age, Blood pressure, Clinical, Duration, Diabetes)*.

† *PAF, Paroxysmal Atrial Fibrillation*.

‡ COPD, Chronic Obstructive Pulmonary Disease;

¥ *WMD, White Matter Disease*.

## Discussion

In our study, the overall six-month rate of the composite outcome (ICH, ischemic stroke, TIA, CAD, and all-caused death) among patients with DWI-neg TIA, DWI-pos TIA, and TIA mimic were 12.2, 9.7, and 2.1%, respectively. The six-month rate of the composite outcome of combined DWI-neg TIA and DWI-pos TIA was 11.7%. More than half of composite outcome events occurred within 3 months following the index event. Patients with TIA mimics had no stroke or ICH event at 6 months follow-up course. Our results highlight the value of a multi-resolution view, where high-fidelity data at multiple time points are used, for better identification of more efficient care plans for this at-risk patient population.

Over the past few decades, there have been multiple studies measuring the rate of stroke following a TIA event. Older studies indicated a range of 9–15% (17–20) within 3 months after a TIA event; however, newer studies reported a lower range of 0.9–4.3% (8, 21–23). In our cohort, patients with DWI-neg TIA had 4.6% risk stroke occurrence at 6 months. Although there has been considerable heterogeneity among studies due to different TIA phenotype and outcome definition, the calculated risk of stroke (4.6%) in our study is similar to recent studies. There are limited literature on TIA mimics; however, we found two studies (3, 8), that evaluated the outcome of patients with a TIA mimics. Our TIA mimics patients had no stroke outcome event at 6 months follow-up course which was similar to the 3 months TIA mimics outcome results in the other two studies (3, 8). Our TIA mimics patients had a composite outcome rate of 2.1% at 6 months including 1.0% all-cause death rate and 1.0% CAD that were closely similar to the retrospective study by Dutta et al. (8) with the all-cause death rate of 0.6% and CAD rate of 0.6% at 3 months follow-up course. It is noteworthy that the lack of a widely approved definition for TIA mimics and heterogeneous nature of TIA mimics should be taken into account for interpretation of outcome in different studies.

The decremental trend of the cerebral ischemia recurrence could be attributed to the changes in the definition of TIA, better secondary prevention, and faster evaluation of TIA patients (24). Recent studies have shown that over the past decade patients with primary TIA or stroke in developed countries are older probably due to improved primary prevention. The concept of rapid access TIA clinic or urgent in-hospital care might have also played an important role in reducing recurrent ischemic events.

In our study, patients with DWI-pos TIA had a 9.7% rate of the composite outcome within 6 months follow-up course. Comparison of our results with 1-year outcome assessment of a large international cohort (22) shows a barely similar composite outcome rate (6.2%). One important difference between these two studies is that we used MRI to differentiate between DWI-pos TIA and DWI-neg TIA while in the other study a mixed pool of DWI-neg TIA or DWI-pos TIA patients were considered for outcome assessment. We did not have any death during the 6-month follow-up course while in the other study all-cause death rate was 1.8%. This difference could be easily attributed to the studied population size and longer follow-up course. The most common single outcome event in our DWI-pos TIA patients was TIA (6.5%) which was similar to the other study result (7.4%).

As it has shown in our study and other studies, patients with DWI-pos TIA or DWI-neg TIA have the highest risk of recurrent ischemic stroke event in the first 30 and 90 days following index event (25), this highlights the importance of rapid and proper evaluation and treatment of the initial ischemic event. The role of hypertension as an important modifiable risk factor for stroke has been known, however, in recent metanalysis hypertension is found to be the most important modifiable risk factor for stroke recurrence, as well (26). In our cohort, we had a meaningful difference among patients with diagnoses of DWI-neg TIA and DWI-neg TIA and patients with a TIA mimic in terms of hypertension. We also observed a higher rate of hypertension (*P* = 0.078) among patients with an outcome event; however, the difference did not reach a significant level. Taken altogether, strict hypertension control along with other modifiable risks management in the early phase after index event could take a unique role in the secondary stroke prevention. Although in our cohort more than 60% of our composite outcome events occurred in the first 90 days, which is consistent with previous studies, we still need to answer an important question about the value of risk factor modification beyond the early follow-up course (27). Finally, the multi-resolution design in this study was important to draw some of these important conclusions.

Although evaluation of the outcome among patients with diagnosis of TIA mimics, considering the heterogenic nature of the condition, is difficult, the very low rate of adverse outcome among patients in this group can be clinically important. It can further highlight the importance of triaging, and proper diagnosis of these patients as many of these patients might not require urgent clinical evaluation and hospitalization. Some of our patients in this group had the alternative diagnoses of toxic or metabolic encephalopathy that encompasses a wide variety of diseases that require a different treatment plan. The main etiology of single death outcome event in this group was cancer, not cerebrovascular disease; however, the most frequently defined alternative diagnosis was migraine similar to a previous report (28). Altogether, it suggests that outcome study in TIA mimics is challenging however it confirms that majority of these patients might not benefit from a hospital admission.

The ABCD^2^ score has been widely used, since its introduction, in the primary care setting for identification of the patients who have a higher risk of stroke recurrence. Although we found a significantly lower ABCD^2^ score among patients with TIA mimics, the score failed to predict the risk of the composite outcome occurrence at 6 months. Our finding is similar to the results of recent studies (29, 30), which showed that the ABCD^2^ score might not be a reliable tool to define a higher risk of the recurrent stroke.

Our study has some limitations. Although the patients were recruited from different hospitals in rural and urban areas in central and northeast Pennsylvania, the study was a single healthcare system study. We had a relatively small sample size with lack of ethnic diversity in the study population. Nevertheless, the lack of diversity could also be considered as a strength of this study due to reduced heterogeneity and higher degrees of generalizability to a similar population.

In conclusion, our study indicated that the overall six-month rate of the composite outcome (ICH, ischemic stroke, TIA, CAD, and all-cause death) among patients with DWI-neg TIA, DWI-pos TIA, and TIA mimics were 12.2, 9.7, and 2.1%, respectively. More than half of the composite outcome events occurred within 90 days following the index event. Age was the only factor that was significantly associated with the occurrence of the composite outcome.

## Data Availability

The datasets generated for this study are available on request to the corresponding author.

## Ethics Statement

This study was part of the ongoing Geisinger stroke registry and approved by institutional review board of Geisinger.

## Author Contributions

ASa: chart review, data gathering, clinical diagnosis, statistical analysis, manuscript writing. VA: data gathering, data archive designer, statistical analysis, manuscript review. ASt, MB, and NH: manuscript review. RZ: study designer, clinical diagnosis, statistical analysis, manuscript writing. NE: chart review, clinical diagnosis.

### Conflict of Interest Statement

The authors declare that the research was conducted in the absence of any commercial or financial relationships that could be construed as a potential conflict of interest.
